# HemoScreen hematology analyzer compared to Sysmex XN for complete blood count, white blood cell differential, and detection of leukocyte abnormalities

**DOI:** 10.1002/jha2.566

**Published:** 2022-09-08

**Authors:** Anna‐Maria Linko‐Parvinen, Kristiina Keränen, Kaisa Kurvinen, Anri Tienhaara

**Affiliations:** ^1^ Tyks Laboratories Clinical Chemistry Turku University Hospital Turku Finland; ^2^ Department of Clinical Chemistry University of Turku Turku Finland

**Keywords:** abnormal leukocytes, comparison, hematology analyzer, HemoScreen

## Abstract

We compared a point‐of‐care HemoScreen hematology analyzer to an automated Sysmex XN analyzer for complete blood count (CBC) and white blood cell (WBC) differential, and evaluated its capacity to detect leukocyte abnormalities. A total of 100 K2‐EDTA whole blood samples, median age 56 years (2 months to 92 years), were compared. For CBC and WBC differential we compared 74 samples with no confirmed abnormal leukocytes. For 26 samples both analyzers gave flagging regarding leukocytes and the accuracy of the flagging was compared. Abnormal leukocytes were confirmed with manual microscopy (200 cells). HemoScreen CBC and WBC differential were highly comparable to Sysmex XN for most of the essential parameters (*r* = 0.909–0.975). More variation was seen for basophil and monocyte counts (*r* = 0.452 and 0.753, respectively). Sysmex XN gave more false WBC abnormal flagging (*n* = 15 altogether) compared to HemoScreen. In addition, Sysmex XN, as well as HemoScreen, gave false WBC flagging for eight samples confirmed normal. The samples verified by microscopy review to truly contain leukocyte abnormalities (*n* = 18) were flagged abnormal with both analyzers. The specificity for analyzer flagging was 72% and 88% for Sysmex XN and HemoScreen, respectively. HemoScreen hematology analyzer is essentially comparable to Sysmex XN for CBC and WBC differential analysis. Most importantly, HemoScreen detected all the samples confirmed to include abnormal leukocytes. HemoScreen was less prone for false WBC flagging compared to Sysmex XN, thereafter requiring less microscopy review. These abilities increase its utility in small health care units. Studies with a larger number of abnormal leukocyte samples are needed to confirm HemoScreen performance.

## 1 INTRODUCTION

An automated hematology analyzer should provide repeatable results for complete blood count (CBC). The measurement range should be wide enough to cover cytopenias and cytoses, and other clinically relevant hematological conditions. In addition, the analyzer's ability to provide reliable white blood cell (WBC) five‐part‐differential and to detect blood cell abnormalities is of high relevance. Samples with possible WBC abnormalities should be reviewed by manual microscopy to correctly identify patients with acute hematological diseases, such as acute leukemia. Other blood cell abnormalities have importance, for example, in diagnosis of the type of anemia.

Most hematology point‐of‐care (POC) devices provide CBC, or parts of CBC, such as hemoglobin concentration, and some give WBC five‐part‐differential [1, 2, 3]. However, their ability to detect blood cell abnormalities is often limited. HemoScreen POC device is a rather novel hematology analyzer, in which the enumeration and identification of blood cells is based on flow cytometry and digital imaging in a single plane using microfluidic viscoelastic focusing 4, 5, 6. The analytical technology combined to artificial intelligence (AI) and computational algorithms identify the cells based on their morphological properties, such as nuclear lobulation, cytoplasmic granulation and cell size. Cells not filling the built‐in algorithms are classified abnormal and the analyzer will give alarms (flagging) concerning these samples [5, 7] The technology based on imaging each cell passing the detector in a plane is shown to achieve high and stable focusing with less interference compared to traditional flow cytometry [5, 6].

Studies on the performance of HemoScreen show that the analyzer is accurate and precise in analyzing red cell parameters compared to Sysmex XN [8]. With intensive care patients, HemoScreen gave good correlation for platelet, WBC and red blood cell (RBC) concentrations compared to Sysmex XN, although the bias was positive at higher platelet and WBC concentrations [9]. Another study with acute leukemia patients showed similar trend for platelets and WBC with a good correlation for complete blood count (CBC), absolute neutrophil count and hemoglobin compared to Sysmex XN [10]. None of these studies investigated the performance on full WBC differential or the analyzer's ability to detect WBC abnormalities, even though there is technology and built‐in algorithms for this.

Sysmex XN analyzer uses impedance with direct current to enumerate platelets and RBCs and to determine RBC indices. For leukocyte enumeration and differentiation Sysmex XN lyses RBCs and uses flow cytometry with a semiconductor laser (633 nm) and detection with forward‐scattered (FSC), side‐scattered (SSC), and side‐fluorescent (SFL) light. The analyzer classifies WBC based on cell size, nuclear polymorphism, cytoplasm granularity and staining intensity. White cell nucleated (WNR) channel with SFL versus FSC is used to enumerate total WBC and basophils. White blood cell differential (WDF) channel with SSC versus SFL differentiates and enumerates neutrophils, eosinophils, lymphocytes, and monocytes, and detects abnormal cells. Fluorescence with FSC vs. SFL can also be used to confirm platelet enumeration specifically in thrombocytopenic samples [11]. AI algorithms further detect possible abnormalities in the cells and may trigger interpretive program messages (flagging) directing the samples to microscopy review. Sysmex XN performance in quantifying blood cells and detecting abnormal blood cells has been evaluated in numerous studies [11, 12, 13, 14, 15, 16, 17, 18].

In this method comparison study, we present the performance of HemoScreen POC device compared to Sysmex XN analyzer. In addition to analytic performance with samples with normal CBC and WBC differential, we focused on the ability of HemoScreen to detect abnormal leukocytes compared to Sysmex XN interpretive program messages and manual microscopy.

## 2 MATERIALS AND METHODS

Within run and total method repeatability were assessed with two level controls (CBC PIX Hematology Controls, ref PIX002, Bio‐Techne Company, lot PIX210605) using a 2 × 2 × 3 protocol, where the controls were analyzed twice in every series twice a day for three consecutive days.

The samples (*n* = 100, with 47 female, median age 56 years [range 2 months to 92 years]) were routine patient K2‐EDTA whole blood samples from outpatient and hospitalized patients, with a request for CBC and WBC differential in 8 May to 2 June 2022. The samples were randomly selected from routine Sysmex XN analyzers to cover a wide variety of CBC and WBC differential results with emphasis on samples with WBC flagging (abnormal and blast cells). The flagging thresholds for Sysmex XN were quantitative and qualitative (low WBC [< 1 ×10^9^/L], left shift, immature granulocytes [limit 3%], atypical/abnormal lymphocytes/blasts, and abnormal WBC/DIFF scatter gram). For Sysmex XN, the flagging thresholds according to the instrument recommendations were used, except for the low WBC count. For HemoScreen, the default flagging thresholds for measurements outside linear ranges and flagging requiring further review (possible nucleated RBC, immature granulocytes, blast cells, atypical lymphocytes and band forms, platelet clumps, abnormal distribution of PLT cell volumes, and low WBC count) were applied. The samples were analyzed within 8 h after drawing the sample first with routine Sysmex XN (Sysmex Co., Kobe, Japan) analyzer and then with HemoScreen (PixCell Medical, Yokneam Ilit, Israel). For microscopy review, the blood films were prepared with Sysmex SP‐50 (Sysmex Co., Kobe, Japan) and dyed with May‐Grünwald‐Giemsa dye (MGG, RAL Diagnostics, Martillac, France). The samples were reviewed with digital microscopy DI‐60/CellaVision (Sysmex Co., Kobe, Japan), or with manual light microscopy, and 200 WBC were reviewed. With microscopy review, WBC differential was reported as blasts, promyelocytes, myelocytes, metamyelocytes, band form and segmented neutrophils, lymphocytes, monocytes, eosinophils, and basophils [19]. Four trained biomedical laboratory scientists, who routinely perform WBC differential in Turku University Hospital, were responsible for the microscopy review. The samples were analyzed anonymous. This study was conducted in accordance with the Declaration of Helsinki (revised in 2013). No patient permission or evaluation from the ethics committee was needed for this method‐comparison study with anonymized samples. Turku Clinical Research Centre gave permission for the study (T12/008/22).

MedCalc 20.019 with Shapiro–Wilk test, Wilcoxon signed‐rank test, and Passing–Bablok linear regression model due to nonnormally distributed data was used for statistical analysis.

## 3 RESULTS

HemoScreen repeatability is presented in Table 1. Of all 100 study samples, 59 gave no Sysmex XN or HemoScreen flagging regarding WBC and were autovalidated. For 15 samples Sysmex XN gave WBC flagging, but HemoScreen gave no flagging and Sysmex XN five‐part WBC differential was reported after microscopy review with no confirmed leukocyte abnormalities. Including these, a total of 74 samples were considered normal and their results were used for HemoScreen method comparison (Figure 1 and Table 2).

**TABLE 1:** HemoScreen analyser repeatability with two control samples (CBC PIX Hematology Controls)

	Control low			Control high		
Analyte	Goal	Mean	Within run/Total CV%	Goal	Mean	Within run/total CV%
WBC (× 10^9^/L)	3.0	2.9	5.8/5.8	8.1	7.9	5.2/5.2
RBC (× 10^12^/L)	2.8	2.8	1.9/2.0	4.7	4.7	1.4/1.4
HGB (g/L)	80	82	1.8/2.6	152	152	1.4/1.8
HCT (%)	20	20	2.0/2.1	38	37	1.3/1.3
PLT (× 10^9^/L)	75	73	3.6/3.6	273	270	2.0/2.0
MCV (fl)	71	70	0.41/0.41	79	79	0.28/0.28
MCH (pg)	28	30	1.2/1.2	32	32	0.65/0.65
MCHC (g/L)	400	420	1.5/1.5	410	410	0.81/0.81
RDW (%)	16	15	0.48/0.67	12	12	0.28/0.55
Neutrophils (× 10^9^/L)	1.5	1.4	7.7/7.7	4.1	3.9	5.0/5.0
Lymphocytes (× 10^9^/L)	1.1	1.2	4.4/4.4	3.1	3.2	7.3/7.3
Monocytes (× 10^9^/L)	0.2	0.2	15/15	0.50	0.52	8.7/8.7
Eosinophils (× 10^9^/L)	0.10	0.096	22/22	0.30	0.24	14/14
Basophils (× 10^9^/L)	0.1	0.012	9.9/9.9	0,1	0.035	5.7/5.7

*Note*: Protocol was 2 × 2 × 3, with control analysis twice in every sample series twice a day for three consecutive days.

WBC, white blood cells; RBC, red blood cells; HGB, hemoglobin; HCT, hematocrit; PLT, platelets; MCV, mean corpuscular volume; MCH, mean corpuscular hemoglobin; MCHC, mean corpuscular hemoglobin concentration; RDW, red blood cell distribution width.

FIGURE 1Comparison of Sysmex XN and HemoScreen analysers for complete blood count parameters and white blood cell differential. Light dotted line, line of equality; solid line, regression; dark dotted lines, confidence intervals; WBC, white blood cells; RBC, red blood cells; HGB, hemoglobin; HCT, hematocrit; PLT, platelets; MCV, mean corpuscular volume; MCH, mean corpuscular hemoglobin; MCHC, mean corpuscular hemoglobin concentration; RDW, red blood cell distribution width

**Figure:**
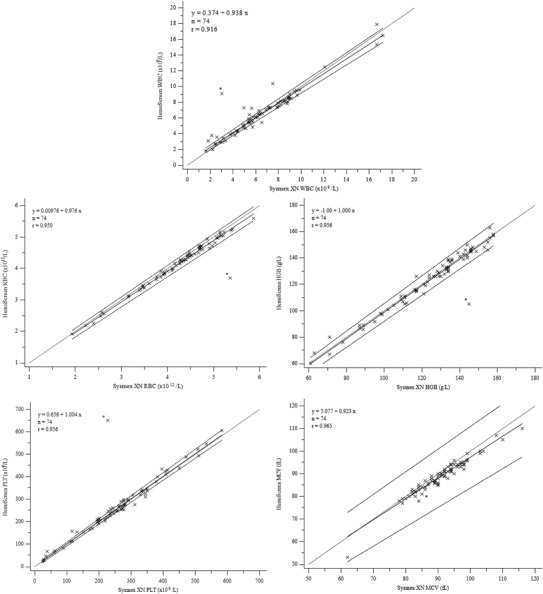

**Figure:**
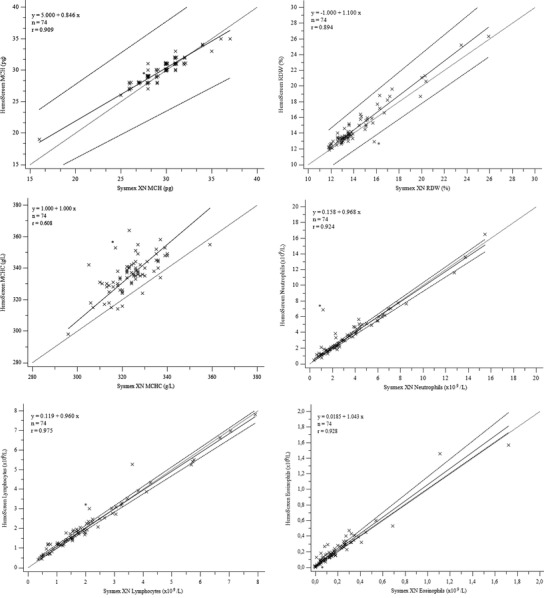

**Figure:**
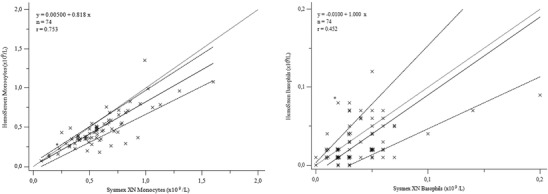

**TABLE 2:** Sysmex XN and HemoScreen complete blood count and white blood cell differential parameters (*n* = 74)

Analyte	HemoScreen median (IQR)	Sysmex XN median (IQR)	*p*
WBC (× 10^9^/L)	6.5 (4.8–8.2)	6.1 (4.9–8.5)	0.8971
RBC (× 10^12^/L)	4.3 (3.7–4.7)	4.4 (3.9–4.7)	<0.0001
HGB (g/L)	128 (109–141)	130 (111–142)	0.0969
HCT (%)	38 (32–41)	40 (35–44)	<0.0001
PLT (× 10^9^/L)	256 (200–323)	260 (198–332)	0.1771
MCV (fl)	89 (84–94)	91 (86–95)	<0.0001
MCH (pg)	30 (28–31)	30 (28–31)	<0.05
MCHC (g/L)	337 (330–343)	324 (318–329)	<0.0001
RDW (%)	13.8 (13.2–15.8)	13.5 (12.9–15.1)	<0.0001
Neutrophils (× 10^9^/L)	3.1 (1.9–4.9)	2.7 (1.8–4.4)	0.2047
Lymphocytes (× 10^9^/L)	1.8 (1.2–2.6)	1.7 (1.1–2.5)	0.0245
Monocytes (× 10^9^/L)	0.43 (0.35–0.55)	0.56 (0.41–0.70)	<0.0001
Eosinophils (× 10^9^/L)	0.17 (0.10–0.29)	0.14 (0.06–0.26)	<0.0001
Basophils (× 10^9^/L)	0.02 (0.01–0.05)	0.03 (0.02–0.05)	<0.05

WBC, white blood cells; RBC, red blood cells; HGB, hemoglobin; HCT, hematocrit; PLT, platelets; MCV, mean corpuscular volume; MCH, mean corpuscular hemoglobin; MCHC, mean corpuscular hemoglobin concentration; RDW, red blood cell distribution width; IQR, interquartile range 25th–75th percentile.

For 26 samples both analyzers gave flagging regarding WBC or HemoScreen did not give any result for CBC or WBC differential. These samples were excluded from automated method comparison but were used to evaluate the congruity of the two analyzers in detecting leukocyte abnormalities. Of these, 18 samples were verified to include abnormal leukocytes with manual microscopy (Table 3). In eight samples both analyzers gave flagging, but the microscopy review showed no abnormal cells and the Sysmex XN result was reported (Table 4). Thereafter, specificity for analyzer flagging was 72% for Sysmex XN and 88% for HemoScreen in these study samples. Sensitivity for autovalidated samples could not be confirmed, as the samples with no flagging were not further reviewed according to routine autovalidation practices.

**TABLE 3:** Samples with Sysmex XN and HemoScreen white blood cell (WBC) flagging, and with confirmed abnormal or immature WBC in manual microscopy (200 cells)

Sample	Sysmex flagging^a^	HemoScreen flagging^b^	Blasts (%)	Promyelocytes (%)	Myelocytes (%)	Metamyelocytes (%)	Band forms (%)	Neutrophils (%)	Lymphocytes (%)	Monocytes (%)	Eosinophils (%)	Basophils (%)
1	1, 2, 3, 4, 8	WBC/Diff^Abn^	5	1	10	4	6	19	39	8	7	2
2	1, 2, 3, 4, 8, 10	Measurement failure	4	0	4	7	5	56	10	10	6	0
3	4, 5, 7, 8	WBC ^Abn^; Diff^	36	0	0	0	0	0	64	0	0	0
4	1, 4	WBC/Diff ^Abn^	78	0	0	0	0	1	19	2	0	0
5	2, 3, 4	WBC/Diff^*^	2	0	5	0	3	46	7	34	3	0
6	1, 2, 4	WBC^HH^; Diff^—^	69	0	1	1	1	20	5	3	1	0
7	1, 2, 4	Diff ^Abn^	39	0	22	0	0	15	17	8	1	0
8	1, 2, 3, 4, 7	WBC/PLT^!^; Diff^Abn^	2	0	10	11	4	35	23	14	1	1
9	1, 2, 3, 4, 7, 8	WBC/Diff ^Abn^	0	0	1	2	7	88	1	1	0	0
10	2, 3	WBC/Diff^*^	0	0	1	11	2	44	7	35	0	1
11	1, 3, 4, 8, 9, 10	Diff ^Abn^	0	0	0	1	4	40	48	6	2	0
12	1, 3, 4, 8, 9	WBC/PLT^!^; Diff^Abn^	0	0	1	1	2	43	50	3	1	0
13	3, 4	Diff^*^	0	0	1	2	1	66	19	7	1	3
14	1, 2, 4	Diff^	0	0	5	5	3	4	82	2	1	0
15	4	Diff^	0	0	7	4	1	16	10	63	0	0
16	3, 4, 5	Diff^*^	0	0	2	1	2	37	11	47	0	1
17^c^	6	WBC/Diff^*^	0	0	0	0	0	91	4	4	0	2
18^d^	1, 4	WBC^HH^; PLT^!^; Diff^—^	0	0	0	0	0	3	95	1	0	0

^a^ Sysmex flagging: ^1^WBC Abn Scattergram; ^2^Left Shift?; ^3^IG Present; ^4^Atypical Lympho?; ^5^Blasts/Abn Lympho?; ^6^Blasts?; ^7^NRBC; ^8^Thrombocytopenia; ^9^PLT ABN Distribution; ^10^PLT Clumps?

^b^ Hemoscreen flagging: ^Abn^The sample may contain the following abnormal cells: nucleated RBCs (NRBCs), immature granulocytes, blast cells, atypical lymphocytes, band forms. Results are not displayed. *The sample may contain the following abnormal cells: nucleated RBCs (NRBCs), immature granulocytes, blast cells, atypical lymphocytes, band forms. Results are displayed. ^Low WBC count (within linear range but less than 2.0 × 10^3^/μl). ^HH^The measured count is outside (above) the linear range. ^—^In case WBC are out of range, the message — will be displayed. ^!^The sample may contain PLT clumps.

^c^ Sample 17: Mature neutrophilia, recovering from neutropenia.

^d^ Sample 18: Mature lymphocytosis, chronic lymphatic leukemia.

**TABLE 4:** Samples with Sysmex XN and HemoScreen white blood cell (WBC) flagging, but with normal WBC in manual microscopy (200 cells)

Sample	Sysmex flagging^a^	HemoScreen flagging^b^
1	3, 4, 5	WBC^LL^; Diff^—^
2	5	Measurement failure
3	8	WBC/Diff^*^
4	1	WBC^*^; Diff^^^
5	4, 5, 8	PLT^LL^; MPV^—^; Diff^^^
6	2, 3	Diff^*^
7	1, 4, 5	Diff^^^
8	1, 4	WBC/PLT^!^; Diff^*^

*Note*: Automated hematology WBC differential was reported.

^a^ Sysmex flagging: ^1^WBC Abn Scattergram; ^2^Left Shift?; ^3^IG Present; ^4^Blasts/Abn Lympho?; ^5^Atypical Lympho?; ^6^Blasts?; ^7^NRBC Present; ^8^Thrombocytopenia; ^9^PLT ABN Distribution; ^10^PLT Clumps?.

^b^ Hemoscreen flagging: ^*^The sample may contain the following abnormal cells: Nucleated RBCs (NRBCs), immature granulocytes, blast cells, atypical lymphocytes, band forms. Results are displayed. ^Low WBC count (within linear range but less than 2.0 × 10^3^/μl). ^LL^The measured count is outside (below) the linear range. ^—^In case WBC are out of range the message — will be displayed. ^!^The sample may contain PLT clumps.

## 4 DISCUSSION

Other studies have described the HemoScreen analyzer to be user‐friendly and suitable for health care units requiring fast results [6]. However, none of the studies compared analyzer's capacity to detect leukocyte abnormalities. This technical ability is of high clinical relevance in patient care and differential diagnosis, as, for example, CBC alone in acute leukemia differential diagnosis may be uninformative.

This study supports the previous findings of HemoScreen analyzer's good repeatability and analytical stability. Data showed slight difference for CBC parameters and WBC differential in samples without any abnormalities. Hemoglobin concentration and platelet count were slightly, but not statistically significantly lower with HemoScreen. For some parameters, such as MCV and HCT, the two analytical methods showed statistically significant difference. In spite of this, these differences in individual samples were minor and the statistical deviation can be considered acceptable in a clinical setting. In general, all parameters were in good agreement to Sysmex. However, there was one sample with deviating CBC results (Figure 1). This sample was from a child 3 months of age, with no apparent clots and WBC 3.1 × 10^9^/L, RBC 5.4 × 10^12^/L, HGB 145 g/L, HCT 46%, and PLT 227 × 10^9^/L with Sysmex XN. Sysmex XN performed well with this sample, yet it is possible that viscosity was high as it can be with neonates and young babies. According to the manufacturer, HemoScreen is suitable for samples from patients above 2 months. The most probable reason for this limitation is generally high hemoglobin and consequently high blood viscosity in younger babies possibly leading to poorer analyzer performance. High sample viscosity can be challenging for any blood cell analyzer detecting single cells, and may have even a higher impact with viscoelastic focusing technology. Compared to, for example, Sysmex XN, sample is not diluted with HemoScreen, but analyzed in a single cartridge where the abnormal fluidity can be prominent. As a consequence, HemoScreen should be used with caution for samples from young children.

WBC differential in the samples with no leukocyte abnormalities, either autovalidated with Sysmex XN or confirmed normal with manual microscopy, showed good correlation and method agreement for neutrophils and lymphocytes. Wider deviation was seen for eosinophil and monocyte measurements, but this deviation can be considered clinically acceptable. Only rarely are absolute eosinophil or monocyte count of crucial clinical relevance in acute care. Should there be suspicion of eosinophilia, monocytosis, or monocytopenia, a higher scale laboratory analyzer, such as Sysmex XN, should be used for precise diagnostic data. Basophil counts showed poor method agreement, but absolute basophil counts are very low and of little clinical relevance for majority of patients. Basophil count is measured on WNR channel, while other WBC subclasses are measured of WDF channel. This may cause part of the deviation for the methods, as in HemoScreen all WBC are measured using the same methodology.

Sysmex XN gave analyzer WBC flagging with a lower threshold compared to HemoScreen, as 15 samples were marked susceptible for leukocyte abnormalities with Sysmex XN, but were confirmed normal and HemoScreen gave no flagging for these. In comparison, for eight samples HemoScreen, together with Sysmex XN, gave WBC flagging, but no abnormalities were confirmed. Sysmex XN algorithms are suitable for laboratories with a high capacity throughput serving wide spectrum of patients, including hematology and children's wards. In this setting, the analyzer should be able to detect even small abnormalities for evaluation by a skilled morphologist to be better safe than sorry. The disadvantage is a relatively high amount of samples needing microscopy review. HemoScreen was less prone to detect leukocyte abnormalities, which can be a good quality in outpatient clinics, small health care units, remote clinics, and even in temporary hospitals. In these, the analyzer should be reliable enough to detect clear leukocyte abnormalities needing immediate confirmation and patient treatment, but robust enough not to require a high capacity of second tier review.

In this study, 18 samples were confirmed to have detectable leukocyte abnormalities in manual microscopy. Sixteen samples had immature WBC, including blasts. These findings are of high clinical importance and should be further inspected. In one sample WBC differential mainly consisted of mature, small lymphocytes related to chronic lymphocytic leukemia (Sample 18), and in another sample (Sample 17) almost all WBC were mature neutrophils produced as a response to neutropenia. Of these 18 samples most were marked abnormal with HemoScreen either with a flagging “Abn” or an asterisk (*). With abnormal cells flagging, the sample may contain nucleated RBC, immature granulocytes (IG), blast cells, atypical lymphocytes, or band forms and the results are not displayed, while with an asterisk, the sample may contain the same abnormal cells but the results are displayed. For one sample with 69% of blasts (Sample 6, Table 3) HemoScreen was not able to perform WBC differential, probably due to high WBC count (139 × 10^9^/L) exceeding linearity range (80 × 10^9^/L). For another sample with 4% blast cells (Sample 2, Table 3), HemoScreen was not able to give any data (measurement failure) and Sysmex XN gave multiple flagging, including suspicion of platelet clumps. Yet no platelet aggregation was confirmed. However, this suggests abnormalities in viscosity possibly interfering HemoScreen analysis. In these two cases, the samples should be analyzed with another analyzer or reviewed by microscopy as no reliable HemoScreen was obtained. No clinically relevant abnormalities were missed with HemoScreen in this study, but HemoScreen results should be interpreted with caution in case of any flagging or when the analyzer is not readily able to perform the analysis possibly due to abnormal viscoelastic properties of a sample.

The weakness of this study is a limited amount of samples and limited variety of leukocyte abnormalities. More comparison studies with wide patients sample material are needed to confirm the performance of HemoScreen analyzer in various clinical conditions. We remain curious to see, whether microfluidic viscoelastic focusing will be incorporated into high capacity automated hematology systems.

## CONFLICT OF INTEREST

The authors declare no conflict of interests.

## FUNDING STATEMENT

The authors received no funding for this study.

## DATA AVAILABILTY STATEMENT

The data that support the findings of this study are available from the corresponding author upon reasonable request.
